# Association of antithrombotic therapy with postoperative rebleeding in patients with cerebral amyloid angiopathy

**DOI:** 10.1186/s41016-023-00324-5

**Published:** 2023-05-01

**Authors:** Taro Yanagawa, Hiroki Sato, Kaima Suzuki, Hidetoshi Ooigawa, Masaki Takao, Hiroki Kurita

**Affiliations:** 1 Department of Cerebrovascular Surgery, International Medical Center, Saitama Medical University, 1397-1 Yamane, Hidaka City, Saitama 350-1298 Japan; 2 Present Address: Stroke Center, Sagamihara Kyodo Hospital, 4-3-1 Hashimotodai, Midori-Ku, , Sagamihara City, Kanagawa-Pref 252-5188 Japan; 3 Department of Clinical Laboratory, National Center of Neurology and Psychiatry, National Center Hospital, 4-1-1, Ogawa-Higashi, Kodaira, Tokyo, 187-8502 Japan

**Keywords:** Amyloid-β, Antithrombotic therapy, Cerebral amyloid angiopathy, Postoperative rebleeding, Subcortical hemorrhage

## Abstract

**Background:**

Cerebral amyloid angiopathy is a common cause of subcortical hemorrhage in older adults. Although open hematoma removal may be performed for severe subcortical hemorrhage, its safety in patients with cerebral amyloid angiopathy has not been established, and postoperative rebleeding may occur. Therefore, this study aimed to investigate factors associated with postoperative rebleeding.

**Methods:**

Out of 145 consecutive patients who had undergone craniotomy for surgical removal of subcortical intracerebral hemorrhage between April 2010 and August 2019 at a single institution in Japan, we examined 109 patients with subcortical hemorrhage who met the inclusion criteria. After excluding 30 patients whose tissue samples were unsuitable for the study, the final study cohort comprised 79 patients.

**Results:**

Of the 79 patients, 50 (63%) were diagnosed with cerebral amyloid angiopathy (cerebral amyloid angiopathy group) and 29 (37%) were not diagnosed with noncerebral amyloid angiopathy (noncerebral amyloid angiopathy group). Postoperative rebleeding occurred in 12 patients (24%) in the cerebral amyloid angiopathy group and in 2 patients (7%) in the noncerebral amyloid angiopathy group. Preoperative prothrombin time–international normalized ratio and intraoperative bleeding volume were significantly associated with postoperative rebleeding in the cerebral amyloid angiopathy group (odds ratio = 42.4, 95% confidence interval = 1.14–1578; *p* = 0.042 and odds ratio = 1.005, 95% confidence interval = 1.001–1.008; *p* = 0.007, respectively).

**Conclusions:**

Patients with cerebral amyloid angiopathy-related cerebral hemorrhage who are receiving antithrombotic therapy, particularly warfarin therapy, are at a high risk of postoperative rebleeding.

**Trial registration:**

Registry and Registration Number of the study: 19–220, 2019/12/23, retrospectively registered.

## Background

Cerebral amyloid angiopathy (CAA) is a well-known cause of cerebral subcortical hemorrhage in older adults and is characterized by the deposition of amyloid-ꞵ (Aβ) protein [1, 2]. Furthermore, CAA has been reported to be associated with Alzheimer’s disease [3, 4], as Aβ protein accumulation in the brain is thought to be involved in the development of both Alzheimer’s disease and CAA [5]. Because some patients with CAA develop cerebral subcortical hemorrhage, several studies on risk factors of cerebral subcortical hemorrhage other than Aβ accumulation have been conducted [6–9].

Craniotomy hematoma removal for CAA-related spontaneous intracerebral hemorrhage (CAA-ICH) has been reported [10, 11]. However, postoperative rebleeding and poor outcomes occurred in some patients, and scientific evidence to support the effectiveness of this treatment strategy is limited [10, 12]. Thus, the present study aimed to investigate factors associated with postoperative rebleeding and treatment outcomes of patients pathologically diagnosed with CAA-ICH who underwent craniotomy hematoma removal.

## Methods

### Study design

We retrospectively collected and analyzed data from consecutive patients who underwent craniotomy hematoma removal for subcortical hemorrhage at Saitama Medical University International Medical Center (SIMC), Japan. CAA was pathologically diagnosed using Aβ staining. Postoperative rebleeding factors were investigated in patients who were pathologically diagnosed with CAA.

### Patient selection

**Fig. 1:**
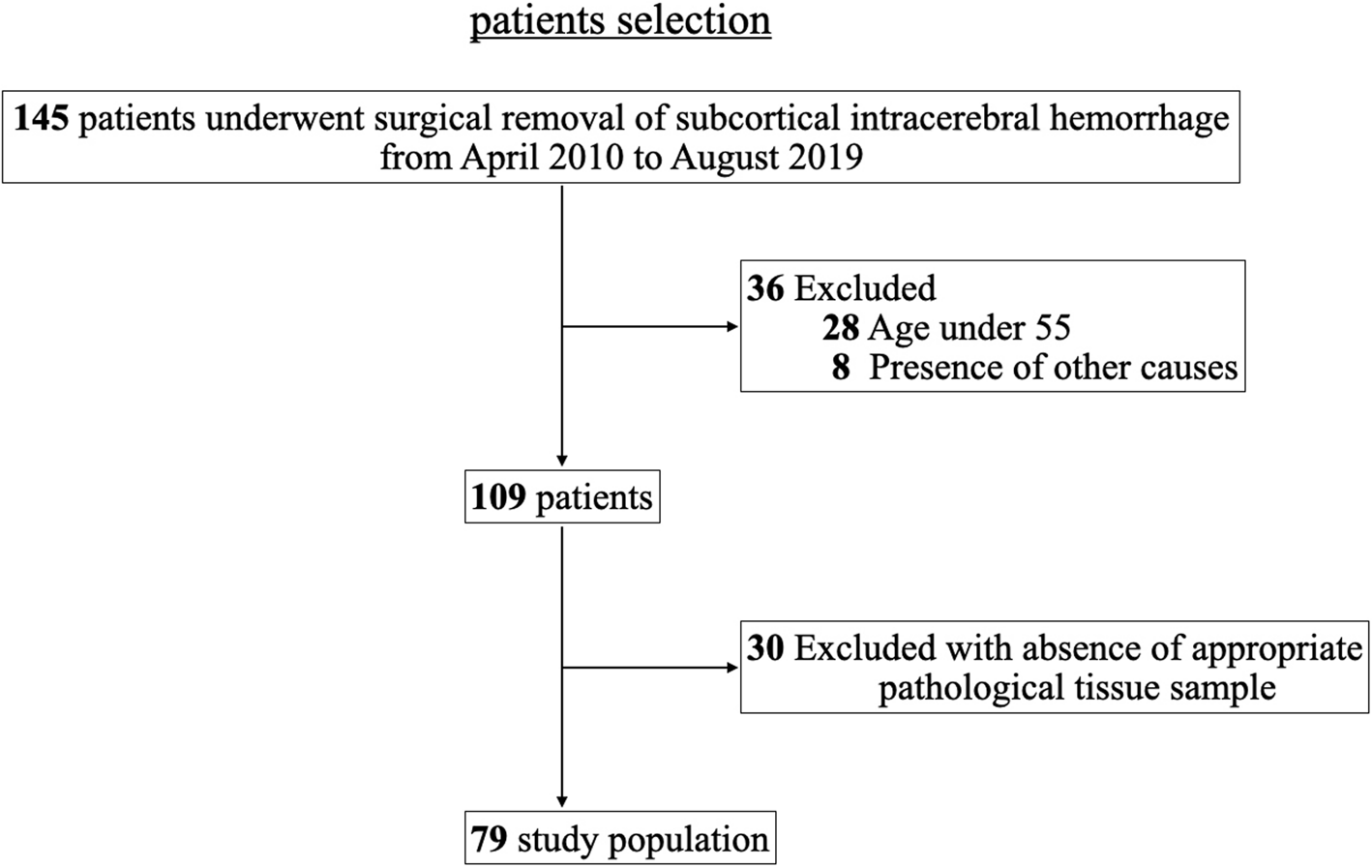
Details of patient selection. Of the 145 patients, 79 met the inclusion criteria. ICH: intracranial hemorrhage

Between April 2010 and August 2019, 145 consecutive patients underwent surgical removal of subcortical intracerebral hemorrhage at SIMC. Subcortical hemorrhage was determined based on radiological findings, and cases of basal ganglia hemorrhage were excluded. Patients who met the following Boston criteria were included in the study: (1) age > 55 years and (2) absence of other causes of hemorrhage. The phrase “other causes” was defined as excessive warfarin intake (i.e., international normalized ratio [INR] > 3.0), antecedent cranial trauma or ischemic stroke, central nervous system tumor, vascular malformation, vasculitis, blood dyscrasia, or coagulopathy [13, 14]. However, since this study aimed to evaluate the complications of antithrombotic medications, patients with excessive warfarin intake and those with coagulopathy were also included in this study. Furthermore, patients with Alzheimer’s disease, other dementias, and hypertension were included in this study. We excluded patients whose tissue samples did not contain any assessable vessels or parenchyma. The final study cohort comprised 79 patients (Fig. 1). Immunohistochemical staining for Aβ was performed with the tissue samples of all 79 patients to determine the presence or absence of CAA. Based on the results of the immunohistochemical staining, patients with the pathological diagnosis of CAA were classified as the CAA group and those without the pathological diagnosis of CAA were classified as the non-CAA group. Monoclonal antibodies specific for Aβ 11–28 (12B8, 1:100, IBL, Gunma, Japan) and Aβ 35–40 (1A10, 1:100, IBL) and polyclonal antibodies specific for Aβ 1–42 (1:100, IBL) were used in the immunohistochemical studies.

### Clinical and neuroimaging data collection

We retrospectively reviewed clinical and neuroimaging data of the enrolled patients from their electronic medical records. The following clinical data were systematically collected for each patient: age; sex; systolic and diastolic blood pressures on admission; hematoma volume; modified Rankin scale (mRS) score at the time of discharge; surgical duration; intraoperative bleeding volume; and history of hypertension, diabetes, dementia (defined as previous diagnosis or current use of antihypertensive, antidiabetic, or antidementia drugs, respectively), or antithrombotic therapy.

Hematoma volume was calculated using the ABC/2 technique, where A is the maximum diameter of the hemorrhagic area, B is the diameter at 90° to A, and C is the number of computed tomography (CT) slices indicating hemorrhage multiplied by the slice thickness [15].

We also assessed the presence or absence of rebleeding within 1 month after craniotomy hematoma removal. Rebleeding after surgery was ascertained by radiologists. Cases of hematoma of volume ≤ 10 mL localized in the resection cavity were excluded.

### Statistical analysis

Age, hematoma volume, systolic and diastolic blood pressures on admission, surgical duration, and intraoperative bleeding volume were analyzed using Student *t*-test. Sex and history of hypertension, diabetes, dementia, and antithrombotic therapy were analyzed using Pearson χ2 test or Fisher exact test. To identify confounding factors, multiple logistic regression analysis was performed using forward stepwise selection methods, with postoperative rebleeding as the outcome. Statistical significance was set at *p* < 0.05. All analyses were performed using SPSS version 26 (IBM Corp., Armonk, NY).

## Results

### Patient characteristics

The clinical characteristics of the total cohort, CAA group, and non-CAA group are presented in Table 1. The median age of the patients included in the study was 76 years (range: 55–89 years). Of the total 79 patients, 41 (52%) were women. There were 40 patients (51%) with hypertension, 17 patients (22%) with dementia, and 30 patients (38%) consuming antithrombotic drugs. In total, 23 patients (29%) were on antiplatelet medications and 11 patients (14%) were on anticoagulants; none of them were on deep vein thrombosis prophylaxis. The mean surgical duration was approximately 185 min, and the mean intraoperative blood loss volume was approximately 308 mL (range: 10–1200 mL).

**Table 1:** The clinical characteristics and postoperative rebleeding rate of patients classified by pathological diagnosis of CAA

	Total cohort (*N* = 79)	CAA (*n* = 50)	Non-CAA (*n* = 29)	*P* value
*Character*				
Age, median (range), year	76(55–89)	78(57–89)	73(55–84)	**0.001**
Female, No. (%)	41(52)	34 (68)	7 (24)	** < 0.001**
Hypertension, No. (%)	40(51)	21 (42)	19 (66)	0.062
Diabetes, No. (%)	7(9)	2 (4)	6 (21)	**0.025**
Dementia, No. (%)	17(22)	15 (30)	2 (7)	**0.022**
Antithrombotic therapy, No. (%)	30(38)	16 (32)	14 (48)	0.229
Antiplatelets, No. (%)	23(29)	11(22)	12(41)	0.078
Anticoagulants, No. (%)	11(14)	5(10)	6(21)	0.162
Combination, No. (%)	4(5)	0	4(14)	**0.016**
Postoperative rebleeding, No. (%)	14(18)	12(24)	2(7)	0.070
Hematoma vol. (before)*1, Mean (SD), mL	82(33)	82(33)	83(33)	0.866
Hematoma vol. (after)*2, Mean (SD), mL	5.9(5.6)	6.2(5.8)	5.2(5.5)	0.430

*CAA* Cerebral amyloid angiopathy

^*^1: Preoperative hematoma volume

^*^2: Residual hematoma volume immediately after surgery

Seven patients received intravenous vitamin K (vit. K) preoperatively, and they were on warfarin. Nine patients received fresh frozen plasma (FFP) transfusion preoperatively, and they were on antithrombotic drugs. Five patients received both vit. K and FFP transfusion (Table 2). The mean prothrombin time (PT)–INR on admission was 2.18 for the 11 patients who received vit. K or FFP and 1.03 for the 67 patients who received neither vit. K nor FFP (*p* = 0.003). Furthermore, the mean activated partial thromboplastin time (APTT) on admission was 42.6 for the patients who received vit. K or FFP and 28.8 for the patients who received neither vit. K nor FFP (*p* = 0.005). Because the surgery was an emergency surgery, few retests were performed after vit. K or FFP administration. In all cases, PT–INR and APTT normalized the day after surgery. All patients underwent standard craniotomy hematoma removal. Minimally invasive procedures, such as endoscopic hematoma removal, were not performed. The CAA group had higher proportions of older adults, women, and patients with dementia but lower proportions of patients with hypertension and those receiving antithrombotic therapy than the non-CAA group.

**Table 2:** The postoperative rebleeding rates for patients who received preoperative Vit. K or FFP

	Total (*N* = 79)	vit.K	FFP	*CAA* (*n* = 50)	vit.K	FFP	*non-CAA* (*n* = 29)	vit.K	FFP
Antithrombotic therapy*,* No. (%)	30(38)	7(9)	9(11)	16 (32)	2(4)	3(6)	14 (48)	5(17)	6(21)
Postoperative rebleeding, No. (%)	14(18)	3(4)	5(6)	12(24)	2(4)	3(6)	2(7)	1(3)	2(7)

*CAA* Cerebral amyloid angiopathy, *vit.K* Intravenous vitamin K, *FFP* Fresh Frozen Plasma

### Rebleeding

Postoperative rebleeding occurred in 14 patients (18%), 12 (86%) of whom were in the CAA group. Rebleeding occurred in three of the seven patients who received intravenous vit. K preoperatively and in five of the nine patients who received FFP transfusion preoperatively (Table 2). Univariate analysis of the CAA group revealed that the proportion of patients on antithrombotic drugs and the volume of intraoperative bleeding were significantly higher in patients who had rebleeding (Table 3). Furthermore, multivariate analysis revealed that postoperative rebleeding was significantly associated with preoperative PT–INR and volume of intraoperative blood loss in the CAA group. Based on the adjusted odds ratio, preoperative PT–INR was strongly associated with postoperative rebleeding (Table 4). The results of Hosmer–Lemeshow test were good (*p* = 0.644), and the percentage of correct classifications was 84.0%.

**Table 3:** Univariate analysis of patients with CAA-ICH classified by the presence of postoperative rebleeding

	Rebleeding( +) (*n* = 12)	Rebleeding(-) (*n* = 38)	*P* value
***Character***			
Age, median (range), year	78.5 (71–86)	78 (57–89)	0.356
Female, No. (%)	10 (83)	24 (63)	0.172
Hypertension, No. (%)	6(50)	15 (39)	0.738
Diabetes, No. (%)	2 (17)	0	0.054
Dementia, No. (%)	4 (33)	11 (29)	0.518
Antithrombotic therapy, No. (%)	7 (58)	9 (24)	**0.032**
Anticoagulant therapy, No. (%)	3 (25)	2 (5)	0.082
Antiplatelet therapy, No. (%)	4 (33)	7 (18)	0.240
***Systemic factor***			
Hematoma vol. (before)*1, Mean (SD), mL	85 (36)	81 (32)	0.721
Hematoma vol. (after)*2, Mean (SD), mL	7 (6)	6 (6)	0.687
Hematoma vol. (rebleed)*3, Mean (SD), mL	28 (14)	6 (6)	** < 0.001**
Systolic blood pressure, Mean (SD), mmHg	184 (25)	173 (34)	0.281
Diastolic blood pressure, Mean (SD), mmHg	88 (30)	84 (22)	0.605
Preoperative PT-INR, Mean (SD)	1.3(0.64)	1.0(0.12)	0.099
Preoperative APTT, Mean (SD)	29(8)	28(4)	0.521
Surgeon’s experience, Mean (SD), year	4.6(1.8)	5.2(2.9)	0.528
Surgical time, Mean (SD), minute	196 (60)	188 (68)	0.731
Intraoperative bleeding volume, Mean (SD), ml	508 (435)	216 (160)	**0.042**
***Outcome***			
Hospital stays, Mean (SD), day	25(20)	31(18)	0.391
mRS 0–3 at the discharge, No. (%)	0	11 (28)	**0.032**
mRS 6 at the discharge, No. (%)	5 (42)	3 (8)	**0.014**

*CAA* Cerebral amyloid angiopathy, *ICH* Intracerebral hemorrhage, *SD* Standard deviation, *mRS* Modified Rankin Scale, *PT-INR* Prothrombin Time-International Normalized Ratio, *APTT* Activated partial thromboplastin time

^*^1: Preoperative hematoma volume, *2: Residual hematoma volume immediately after surgery, *3: Hematoma volume after rebleeding

The mRS score 0 is no symptoms at all, 1 is no significant disability despite symptoms, 2 is slight disability, 3 is moderate disability, 4 is moderately severe disability, 5 is severe disability, and 6 is death

**Table 4:** Multivariate analysis of factors associated with postoperative rebleeding in CAA-ICH group

	B	*P* value	**Adjusted odds ratio (95%CI)**
Preoperative PT-INR	3.746	0.042	**42.369 (1.137–1578.683)**
Intraoperative bleeding volume	0.005	0.007	**1.005 (1.001- 1.008)**
Statistical Constant Term	-6.871	0.002	**0.001**

*CAA* Cerebral amyloid angiopathy, *ICH* Intracerebral hemorrhage, *B* Partial regression coefficient, *PT-INR* Prothrombin Time-International Normalized Ratio

In total, 10 patients in the CAA group and 2 patients in the non-CAA group presented with rebleeding immediately at the surgical site. Rebleeding was determined based on the comparison of CT images taken immediately after surgery with CT images taken the following day. Two patients in the CAA group had delayed rebleeding at sites distant from the surgical site.

### Outcome

**Fig. 2:**
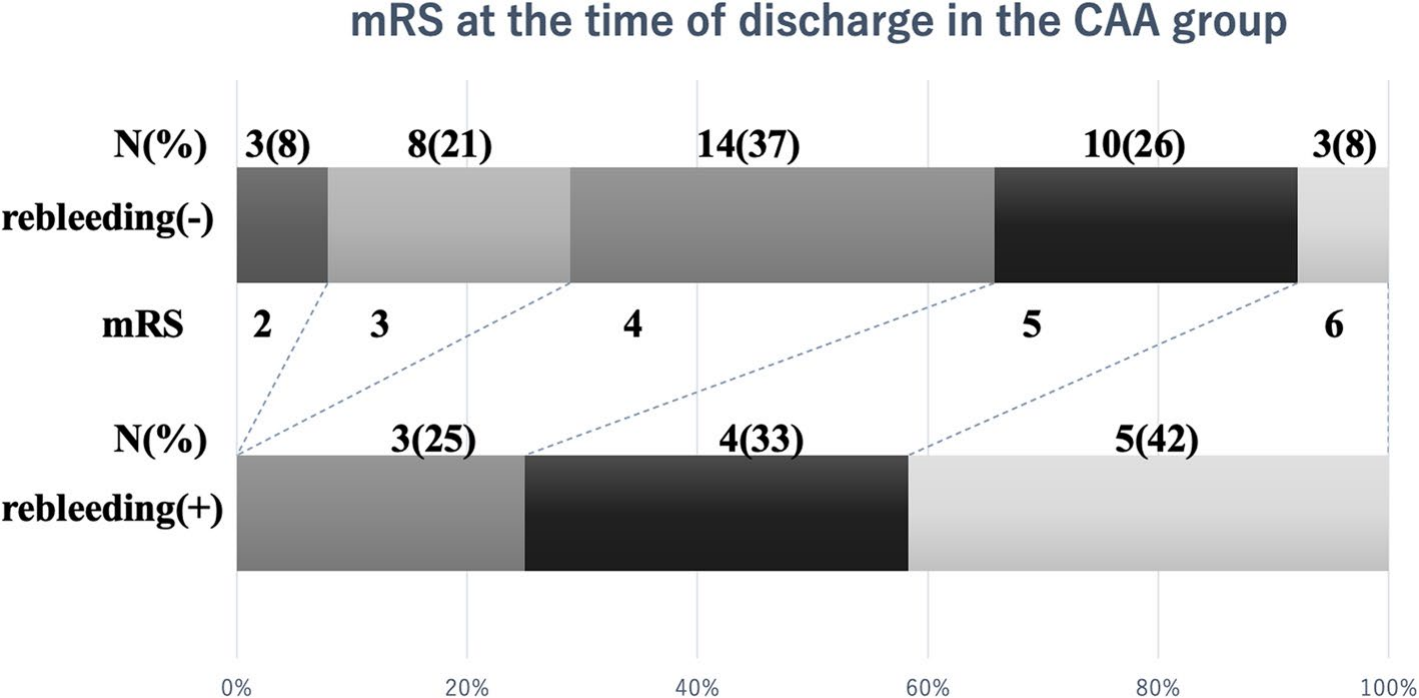
mRS scores of the CAA group at the time of discharge. mRS scores of patients in the CAA group with or without postoperative rebleeding at the time of discharge. An mRS score of 0 indicates no symptoms at all, a score of 1 indicates no significant disability despite symptoms, a score of 2 indicates mild disability, a score of 3 indicates moderate disability, a score of 4 indicates moderately severe disability, a score of 5 indicates severe disability, and a score of 6 indicates death. mRS: Modified Rankin scale

In the CAA group, the mRS score at the time of discharge was 0–3 in 11 of the 50 patients (22%), and the 1-month mortality rate was 16% (8 of the 50 patients). In patients with CAA who experienced rebleeding, the mRS score at the time of discharge was > 3 in all 11 patients, and the 1-month mortality rate was 45% (5 of the 11 patients). In contrast, in patients with CAA who did not have rebleeding, the mRS score at the time of discharge was 0–3 in 11 of the 39 patients (28%), and the 1-month mortality rate was 8% (3 of the 39 patients) (Fig. 2 and Table 3).

## Discussion

This study aimed to identify the risk factors associated with rebleeding after craniotomy hematoma removal in patients with CAA-ICH. Although the results of neurosurgical evacuation for severe CAA-ICH have been reported, the effectiveness of surgical procedures remains unclear.

The incidence rate of postoperative rebleeding in patients pathologically diagnosed with CAA and subcortical hemorrhage varies from 22 to 50%, as reported in previous studies [12, 16, 17]. In our study, 12 out of 50 patients (24%) pathologically diagnosed with CAA had postoperative rebleeding. Petridis et al. showed that patients with CAA-ICH had a significantly higher rate of postoperative rebleeding than patients without CAA-ICH. They also observed that patients with CAA-ICH were significantly older and had a higher level of vascular vulnerability, which increases the risk of rebleeding, than patients without CAA-ICH [12].

In this study, preoperative PT–INR and intraoperative blood loss volume were significantly associated with postoperative rebleeding in the CAA group. The high intraoperative bleeding volume observed in this study (and in other studies) suggests that patients with CAA are older and have more fragile vessel walls than patients without CAA, which makes hemostasis more difficult [12, 18].

Although high preoperative PT–INR is naturally associated with a high risk of bleeding, all patients with high PT–INR received preoperative reversal therapy. It has been reported that early and aggressive anticoagulation recovery therapy and good blood pressure control lead to better outcomes in patients with ICH [19, 20]. However, in the CAA group, anticoagulation recovery therapy may have not been effective in preventing postoperative rebleeding. Due to the small number of cases, we could not comprehensively investigate this theory. Furthermore, the only antithrombotic antagonists used in this study were vit. K and FFP. Our results may have been different if other antagonists (such as four-factor prothrombin complex concentrates or platelet transfusions) had been used, but their efficacy against CAA-ICH remains unknown. In this study, no detection index of antithrombotic therapy other than PT–INR and APTT were evaluated, making the analysis difficult.

Furthermore, reports of the timing and location of postoperative rebleeding are rare. In this study, the 12 cases of immediate rebleeding at the same site highlight the difficulty of complete hemostasis at the surgical site in patients with CAA. In addition, in the two cases of delayed hemorrhage in other areas, postoperative stress and blood pressure fluctuations may have caused the collapse of cerebral vessels in areas that were originally vulnerable. Rebleeding of this type is difficult to prevent.

Our results showed that prognosis was significantly poor and mortality rate was high in patients with CAA when rebleeding occurred after craniotomy hematoma removal (Fig. 2). In other words, if a patient with CAA undergoing antithrombotic therapy has severe cerebral hemorrhage, craniotomy is unlikely to improve prognosis, and there is a relatively high chance that the patient will not survive. Hence, it is critical to consider the surgical strategy for hemostasis and carefully determine the surgical indications for hematoma removal in patients with CAA-ICH who are receiving antithrombotic therapy. Furthermore, antithrombotic therapy should be administered with caution to patients with CAA who are at a high risk of cerebral hemorrhage. For example, as much as possible, warfarin therapy should be avoided for patients with CAA. It is necessary to conduct additional research to develop a simple method for diagnosing patients with CAA and assessing their risk of cerebral hemorrhage.

This study has several limitations. First, this was a single-center study with a small number of patients. Therefore, the generalizability of our results for multiple institutions is limited, and studies with larger sample sizes should be conducted in the future to verify the findings of this study. Second, due to insufficient data, long-term outcomes could not be investigated. Nevertheless, the findings of this study are expected to contribute to a better understanding of the establishment of a treatment strategy for patients with CAA-ICH.

## Conclusion

Craniotomy should be carefully considered for patients with CAA-ICH who are receiving antithrombotic medications and have a high PT–INR because they are at a high risk of postoperative rebleeding. Furthermore, antithrombotic therapy, particularly warfarin therapy, should be administered with caution to patients with CAA.

## Data Availability

The datasets used and/or analyzed during the current study are available from the corresponding author on reasonable request.

## Abbreviations

CAA: Cerebral amyloid angiopathy
Aβ: Amyloid-ꞵ protein
ICH: Spontaneous intracerebral hemorrhage
CAA-ICH: Cerebral amyloid angiopathy-related spontaneous intracerebral hemorrhage
SIMC: Saitama Medical University International Medical Center
mRS: Modified Rankin scale
